# Supplementary material for: Impact of a Rounding Checklist Implementation in the Trauma Intensive Care Unit on Clinical Outcomes
Source: Healthcare (Basel). 2024 Apr 23;12(9):871. doi: 10.3390/healthcare12090871 (PMC11083085; doi:10.3390/healthcare12090871)
Supplement: Supplementary file 1 [file healthcare-12-00871-s001.zip › healthcare-2930856-supplementary.pdf]

AJOU TRAUMA

CHAPTER 1

Principles of Critical Care & Preventive Practices in the TICU: FAST HUGS BID

경기남부 권역외상센터 AJOU TRAUMA CENTER

AJOU TRAUMA CENTER
경기남부 권역외상센터

INTRODUCTION

- Purpose of Care in the ICU ?
  - To reestablish homeostasis
  - To minimize secondary and iatrogenic injury

- Excluding early deaths in the operating room, most traumatic hospital deaths will occur in the ICU.

INTRODUCTION

- A modern surgical ICU in the 21st century
  - Evidence-based care
    - using algorithms, CPGs, and checklists
  - Cutting-edge technology for physiologic monitoring
  - A robust QI process
    - To evaluate its outcomes
    - To identify OFIs

CPGs, clinical practice guidelines, QI; quality improvement, OFIs; opportunities for improvement

ORGANIZATION OF THE ICU

- Patterns of ICU physician organization
  - Closed / 폐쇄형
  - Open / 개방형
  - Semiopen / 통합형

ORGANIZATION OF THE ICU

- **Closed unit / 폐쇄형**
  - Relies almost exclusively on a critical care team (or attending intensivist) for primary management
  - responsibility for all orders and procedures
  - other services providing care as consultants on an as-needed basis
  - 중환자관리를 전공한 의료진 또는 중환자실만을 전담하는 의료진이 주치의가 되어 24시간의 중환자관리가 이루어지는 방식

ORGANIZATION OF THE ICU

- **Open unit / 개방형**
  - a designated ICU director
  - a separate ICU team
  - an intensivist immediately available
  - 중환자실 전담의는 주치의의 자문의로서 특정분야, 호흡기, 영양, 순환기 등의 문제를 담당하며 주치의는 아니지만 항상 24시간 동안 진료를 제공.

ORGANIZATION OF THE ICU

- **Semiopen unit / 통합형**
  - Collaborative plan of practice
  - Intensivists who coordinate care with primary surgeons
  - Primary surgeons may write orders on their patients
  - Critical care team involvement with each patient is typically either mandatory or expected

ORGANIZATION OF THE ICU

- **High-intensity model**
  - 24/7 dedicated physician staffing
  - Mandatory ICU team involvement
  - All closed units / most semiopen units
- **Low-intensity model**
  - Open unites

- Dedicated, higher-intensity intensivist staffing for ICU patients will ultimately **improve outcomes** from a variety of conditions and illnesses

ORGANIZATION OF THE ICU

AJOU TRAUMA CENTER
경기남부 권역외상센터

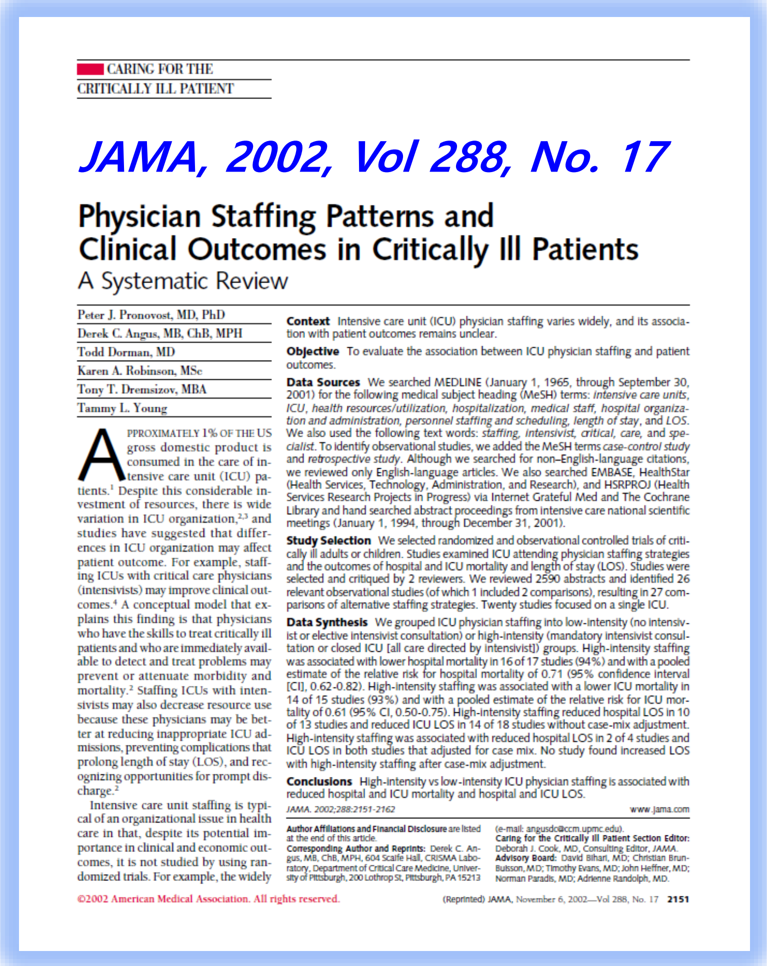

CARING FOR THE CRITICALLY ILL PATIENT

JAMA, 2002, Vol 288, No. 17

Physician Staffing Patterns and Clinical Outcomes in Critically Ill Patients
A Systematic Review

Peter J. Pronovost, MD, PhD
Derek C. Angus, MB, ChB, MPH
Todd Dorman, MD
Karen A. Robinson, MSc
Tony T. Dremsizov, MBA
Tammy L. Young

Context Intensive care unit (ICU) physician staffing varies widely, and its association with patient outcomes remains unclear.

Objective To evaluate the association between ICU physician staffing and patient outcomes.

Data Sources We searched MEDLINE (January 1, 1965, through September 30, 2001) for the following medical subject heading (MeSH) terms: *intensive care units, ICU, health resources/utilization, hospitalization, medical staff, hospital organization and administration, personnel staffing and scheduling, length of stay*, and *LOS*. We also used the following text words: *staffing, intensivist, critical, care*, and *specialist*. To identify observational studies, we added the MeSH terms *case-control study* and *retrospective study*. Although we searched for non–English-language citations, we reviewed only English-language articles. We also searched EMBASE, HealthStar (Health Services, Technology, Administration, and Research), and HSRPROJ (Health Services Research Projects in Progress) via Internet Grateful Med and The Cochrane Library and hand searched abstract proceedings from intensive care national scientific meetings (January 1, 1994, through December 31, 2001).

Study Selection We selected randomized and observational controlled trials of critically ill adults or children. Studies examined ICU attending physician staffing strategies and the outcomes of hospital and ICU mortality and length of stay (LOS). Studies were selected and critiqued by 2 reviewers. We reviewed 2590 abstracts and identified 26 relevant observational studies (of which 1 included 2 comparisons), resulting in 27 comparisons of alternative staffing strategies. Twenty studies focused on a single ICU.

Data Synthesis We grouped ICU physician staffing into low-intensity (no intensivist or elective intensivist consultation) or high-intensity (mandatory intensivist consultation or closed ICU [all care directed by intensivist]) groups. High-intensity staffing was associated with lower hospital mortality in 16 of 17 studies (94%) and with a pooled estimate of the relative risk for hospital mortality of 0.71 (95% confidence interval [CI], 0.62-0.82). High-intensity staffing was associated with a lower ICU mortality in 14 of 15 studies (93%) and with a pooled estimate of the relative risk for ICU mortality of 0.61 (95% CI, 0.50-0.75). High-intensity staffing reduced hospital LOS in 10 of 13 studies and reduced ICU LOS in 14 of 18 studies without case-mix adjustment. High-intensity staffing was associated with reduced hospital LOS in 2 of 4 studies and ICU LOS in both studies that adjusted for case mix. No study found increased LOS with high-intensity staffing after case-mix adjustment.

Conclusions High-intensity vs low-intensity ICU physician staffing is associated with reduced hospital and ICU mortality and hospital and ICU LOS.

JAMA. 2002;288:2151-2162 www.jama.com

APPROXIMATELY 1% OF THE US gross domestic product is consumed in the care of intensive care unit (ICU) patients.[1] Despite this considerable investment of resources, there is wide variation in ICU organization,[2,3] and studies have suggested that differences in ICU organization may affect patient outcome. For example, staffing ICUs with critical care physicians (intensivists) may improve clinical outcomes.[4] A conceptual model that explains this finding is that physicians who have the skills to treat critically ill patients and who are immediately available to detect and treat problems may prevent or attenuate morbidity and mortality.[2] Staffing ICUs with intensivists may also decrease resource use because these physicians may be better at reducing inappropriate ICU admissions, preventing complications that prolong length of stay (LOS), and recognizing opportunities for prompt discharge.[2]

Intensive care unit staffing is typical of an organizational issue in health care in that, despite its potential importance in clinical and economic outcomes, it is not studied by using randomized trials. For example, the widely

Author Affiliations and Financial Disclosure are listed at the end of this article.
Corresponding Author and Reprints: Derek C. Angus, MB, ChB, MPH, 604 Scaife Hall, CRISMA Laboratory, Department of Critical Care Medicine, University of Pittsburgh, 200 Lothrop St, Pittsburgh, PA 15213 (e-mail: angusdc@ccm.upmc.edu).
Caring for the Critically Ill Patient Section Editor: Deborah J. Cook, MD, Consulting Editor, *JAMA*.
Advisory Board: David Bihari, MD; Christian Brun-Buisson, MD; Timothy Evans, MD; John Heffner, MD; Norman Paradis, MD; Adrienne Randolph, MD.

©2002 American Medical Association. All rights reserved.
(Reprinted) JAMA, November 6, 2002—Vol 288, No. 17 **2151**

Meta-analysis of **26** pooled studies

- **Higher-intensity ICU staffing**
  - A relative risk of **0.71** for hospital mortality (95% CI = 0.62-0.82)
  - A relative risk of **0.61** for ICU mortality (95% CI = 0.5-0.75)

ORGANIZ

AJOU TRAUMA CENTER
경기남부 권역외상센터

Prospe … auma centers

- the ef … g
  … major trauma
  - A re … 04)
  - In d … .46–0.88)

TABLE 4. Adjusted Relative Risk of Death in Intensivist-Model ICUs*

	Relative Risk (95% CI)	P (Interaction Term)†
All patients	0.78 (0.58–1.04)	NA
Age		<0.01
<55 yr	1.08 (0.78–1.49)	
≥55 yr	0.55 (0.39–0.77)	
Head injury severity (head AIS)		0.14
<3	0.64 (0.45–0.92)	
≥3	0.85 (0.62–1.16)	
Injury Severity Score		0.37
<25	0.71 (0.49–1.04)	
≥25	0.84 (0.62–1.15)	
Mechanism of injury		0.81
Blunt	0.78 (0.58–1.05)	
Penetrating	0.87 (0.38–1.98)	
Trauma center designation status		<0.01
Trauma center	0.64 (0.46–0.88)	
Nondesignated center	1.42 (0.91–2.22)	
Director is board certified in surgery and critical care		<0.01
Yes	0.67 (0.50–0.90)	
No	1.61 (0.95–2.72)	

ORGANIZATION OF THE ICU

AJOU TRAUMA CENTER
경기남부 권역외상센터

- **Failure to Rescue ?** - the mortality rate among surgical patients who develop complications

Journal of Critical Care 29 (2014) 930–935

Contents lists available at ScienceDirect

Journal of Critical Care

journal homepage: www.jccjournal.org

ELSEVIER

The association of intensivists with failure-to-rescue rates in outlier hospitals: Results of a national survey of intensive care unit organizational characteristics☆

CrossMark

Elliot Wakeam, MD [a,b,*], Denise Asafu-Adjei, BS, MPH [a], Stanley W. Ashley, MD [a], Zara Cooper, MD, MSc [a], Joel S. Weissman, PhD [a,c]

[a] *Center for Surgery and Public Health, Department of Surgery, Brigham and Women's Hospital, Boston, MA*
[b] *Department of Surgery, University of Toronto, Toronto, Canada*
[c] *Patient-Centered Comparative Effectiveness Research Center, Brigham and Women's Hospital, Boston, MA*

ARTICLE INFO

Keywords:
Failure to rescue quality
Safety
Rapid response
Organizational behavior
Critical care

ABSTRACT

Purpose: Critical care is often an integral part of rescue for patients with surgical complications. We sought to understand critical care characteristics predictive of failure-to-rescue (FTR) performance at the hospital level.
Methods: Using 2009 to 2011 FTR data from Hospital Compare, we identified 144 outlier hospitals with significantly better/worse performance than the national average. We surveyed intensive care unit (ICU) directors and nurse managers regarding physical structures, patient composition, staffing, care protocols, and rapid response teams (RRTs). Hospitals were compared using descriptive statistics and logistic regression.
Results: Of 67 hospitals completing the survey, 56.1% were low performing, and 43.9% were high performing. Responders were more likely to be teaching hospitals (40.9% vs 25.0%; $P = .05$) but were similar to nonresponders in terms of size, region, ownership, and FTR performance. Poor performers were more likely to serve higher proportions of Medicaid patients (68.4% vs 20.7%; $P < .0001$) and be level 1 trauma centers (55.9% vs 25.9%; $P = .02$). After controlling for these 2 characteristics, an intensivist on the RRT (adjusted odds ratio, 4.27; confidence interval, 1.45-23.02; $P = .005$) and an internist on staff in the ICU (adjusted odds ratio, 2.13; $P = .04$) were predictors of high performance.
Conclusions: Intensivists on the RRT and internists in the ICU may represent discrete organizational strategies for improving patient rescue. Hospitals with high Medicaid burden fare poorly on the FTR metric.
© 2014 Elsevier Inc. All rights reserved.

J Crit Care 29 (2014), 930-935

EAST 2014 PLENARY PAPER

J Trauma Acute Care Surg. 2014;77: 83-88.

Higher surgical critical care staffing levels are associated with improved National Surgical Quality Improvement Program quality measures

Christopher R. Reed, BS, Sandy L. Fogel, MD, Bryan R. Collier, DO, Eric H. Bradburn, DO, Christopher C. Baker, MD, *and* **Mark E. Hamill, MD,** *Roanoke, Virginia*

BACKGROUND: The changing face of American health care demands careful scrutiny of resource allocation. The impact of the surgical intensivist model on general surgical quality measures has not been studied. Our objective was to investigate the relationship between surgical critical care staffing and indicators of general surgical quality measured by the National Surgical Quality Improvement Program (NSQIP).

METHODS: We retrospectively examined the number of attending surgical intensivists at our tertiary care center biannually from January 2008 through June 2012. Risk-adjusted indicators of general surgical quality were captured and reported semiannually by NSQIP. Mortality, overall morbidity, patients on ventilator for more than 48 hours, unplanned intubations, and venous thromboembolism were included. Student's *t* test was used to compare the staffing levels and associated NSQIP odds ratios of a 3-year control period of full commitment with a 2-year period following significant provider attrition.

RESULTS: The number of full-time surgical intensivists ranged from 2 to 8, with a period of rapid decline in late 2010 to early 2011 followed by slow recovery. There was a mean of 6.6 surgical intensivists during the 3 years before the decline and a mean of 4 in the 2 years after the decline and recovery ($p < 0.005$). This period of decline was associated with a significant increase in the odds ratio of ventilation for more than 48 hours (before, 0.936; after, 1.87; $p = 0.0086$) and of venous thromboembolism (before, 0.844; after 1.43; $p = 0.0268$). A trend in increased unplanned intubations was also observed. Overall morbidity and mortality were not affected. Notably, quality indicators seemed to rapidly approach baseline levels as new surgical intensivists were recruited.

CONCLUSION: Institutional commitment to recruitment and retention of a surgical critical care team leads to improved NSQIP general surgery quality measures. (*J Trauma Acute Care Surg*. 2014;77: 83–88. Copyright © 2014 by Lippincott Williams & Wilkins)

LEVEL OF EVIDENCE: Care management study, level IV.

KEY WORDS: NSQIP; surgical critical care; quality improvement; intensivist model.

QI IN THE ICU

- **Complex process …**
  - Ongoing **identification** of outcome measure or performance indicators
  - **Data collection and analysis**
  - **Development of action plans** to correct deficiencies
  - **Subsequent monitoring** of the performance (outcome) measures

QI IN THE ICU

AJOU TRAUMA CENTER
경기남부 권역외상센터

- **The effectiveness of CPGs** has been demonstrated…
  - Ventilator weaning
  - Pneumonia
  - Nutrition
  - Sedation
  - ….

- **Difficulties…**
  - More to the **implementation…** > development

FAST HUGS BID

AJOU TRAUMA CENTER
경기남부 권역외상센터

Continuing Medical Education

Give your patient a fast hug (at least) once a day*

Jean-Louis Vincent, MD, PhD, FCCM

LEARNING OBJECTIVES

On completion of this article, the reader should be able to:

1. Interpret the mnemonic "Fast Hug."
2. Explain the elements of "Fast Hug."
3. Use this knowledge in a clinical setting.

The author has disclosed that he has no financial relationships or interests in any commercial companies pertaining to this educational activity.

Wolters Kluwer Health has identified and resolved any faculty conflicts of interests regarding this educational activity.

Visit the *Critical Care Medicine* Web site (www.ccmjournal.org) for information on obtaining continuing medical education credit.

Objective: To introduce the Fast Hug mnemonic (Feeding, Analgesia, Sedation, Thromboembolic prophylaxis, Head-of-bed elevation, stress Ulcer prevention, and Glucose control) as a means of identifying and checking some of the key aspects in the general care of all critically ill patients.

Design: Not applicable.

Setting: Any intensive care unit at any time.

Patients: All intensive care unit patients.

Interventions: Dependent on the results of applying the Fast Hug.

Measurements and Main Results: Not applicable.

Conclusions: Application of this simple strategy encourages teamwork and may help improve the quality of care received by our intensive care unit patients. (Crit Care Med 2005; 33:1225–1229)

KEY WORDS: feeding; sedation; analgesia; stress ulcer prevention; semirecumbent; glucose control; thromboembolism

Crit Care Med 2005 Vol. 33, No. 6

Critically ill patients need "FAST HUGS BID" (an updated mnemonic)

To the Editor:

We have recently re
lent article by Vincent
daily "FAST HUG" chec
veloped to highlight th
care for all critically i
used by the multidiscip
We propose an upd
"FAST HUGS BID," wh
assessment for ventilat
a spontaneous breathin
ation and maintenanc
bowel function (B), rem
catheters (I), and de-esc
timicrobial and other p
(Table 1). Although we
this checklist at least
BID), systematic appro
care should, ideally, b
throughout work shifts

Table 1. Components of "FAST HUGS BID"

F	Feeding
A	Analgesia
S	Sedation
T	Thromboembolic prophylaxis
H	Head of bed elevation
U	Ulcer (stress) prophylaxis
G	Glycemic control
S	Spontaneous breathing trial
B	Bowel regimen
I	Indwelling catheter removal
D	De-escalation of antibiotics

Crit Care Med 2009 Vol. 37, No. 7

FAST HUGS BID

Table 1. Components of "FAST HUGS BID"

F	Feeding
A	Analgesia
S	Sedation
T	Thromboembolic prophylaxis
H	Head of bed elevation
U	Ulcer (stress) prophylaxis
G	Glycemic control
S	Spontaneous breathing trial
B	Bowel regimen
I	Indwelling catheter removal
D	De-escalation of antibiotics

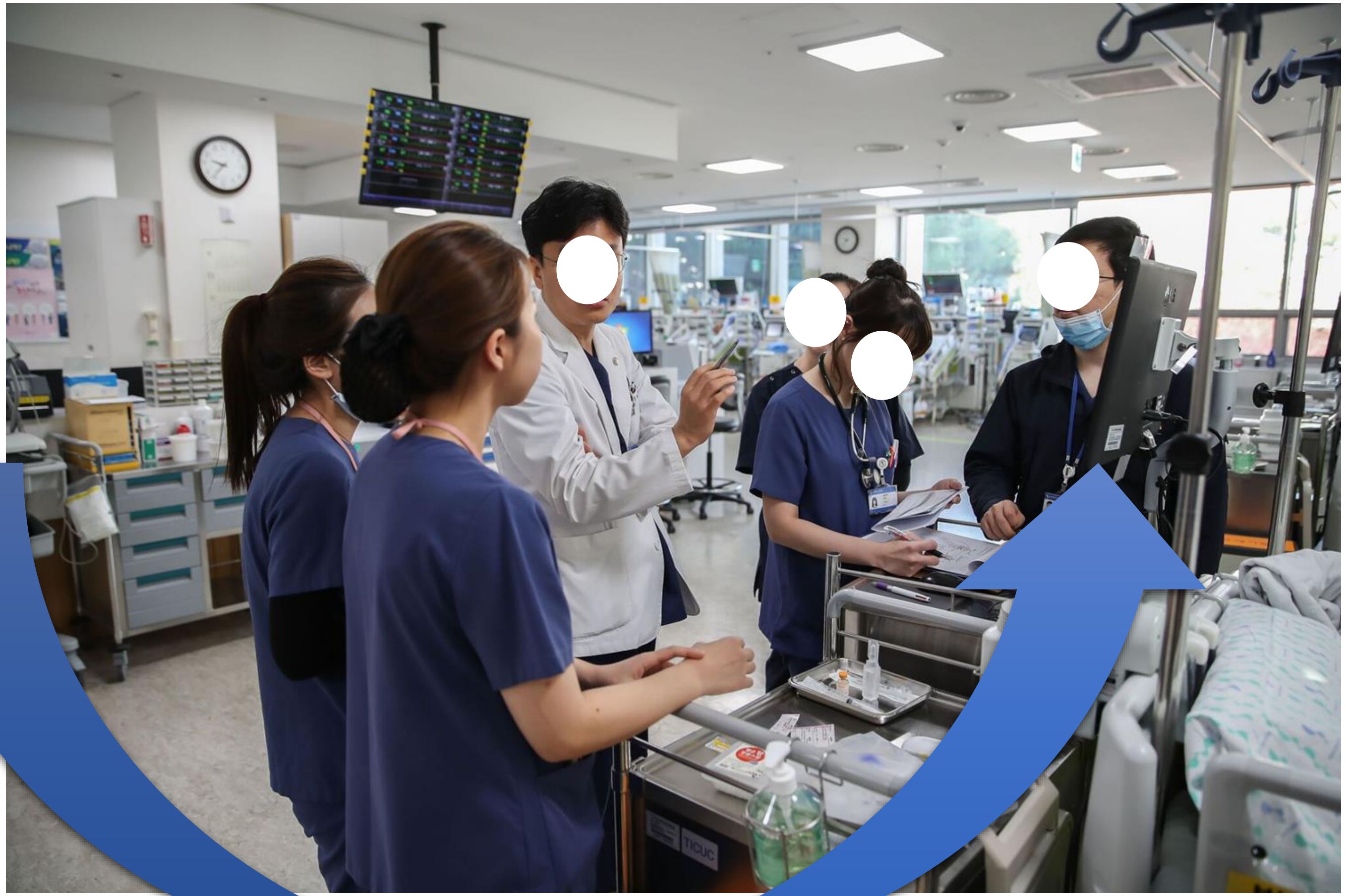

FAST HUGS BID

- ## FEEDING
  - Malnutrition increases complications and worsens outcomes for critically ill patients.
  - If oral feeding is not possible, enteral nutrition is preferred to parenteral nutrition and **should be started early, preferably within 24–48 hours of ICU admission**.
  - The optimal constituents of feeding solutions remain under debate.

FAST HUGS BID

AJOU TRAUMA CENTER
경기남부 권역외상센터

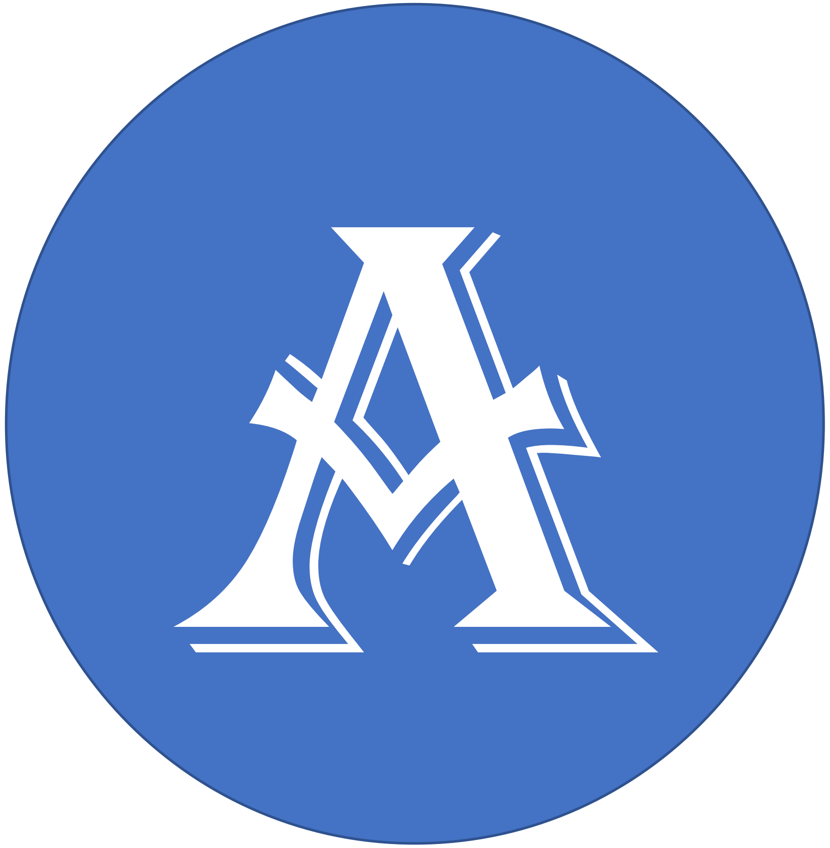

• ANALGESIA

- Pain can affect a patient's psychological and physiologic **recovery**, and adequate pain relief must form an integral part of good intensive care management.
- Critically ill patients feel pain due not only to their illness but also to routine procedures such as turning, suctioning, and dressing changes

FAST HUGS BID

- **SEDATION**
  - Although it may be easier to increase the dose of sedative to have a calm and quiet patient, **oversedation** is associated with harmful effects.
  - Including an **increased risk** of
    - venous thrombosis
    - decreased intestinal motility
    - hypotension
    - reduced tissue O_2 extraction capabilities
    - ICU polyneuropathy
    - prolonged ICU stay
    - increased costs

FAST HUGS BID

- **THROMBOEMBOLIC PROPHYLASIX**
  - Among patients who do not receive prophylaxis, objectively confirmed rates of DVT range between 13% and 31%; for trauma patients this figure may be considerably higher.
  - Clearly, the benefit of prophylaxis must be weighed against the risk of bleeding complications.

FAST HUGS BID

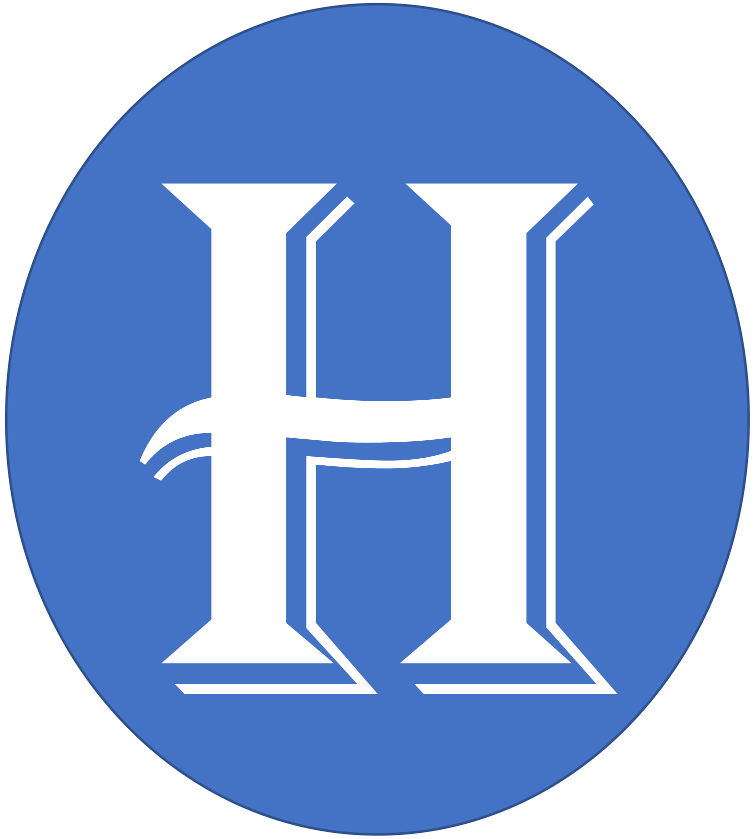

- **HEAD OF BED ELEVATION**
  - Several studies have demonstrated that having the head of the bed inclined at 45 degrees can decrease the incidence of gastroesophageal reflux in mechanically ventilated patients.
  - one randomized, controlled study demonstrated reduced rates of nosocomial pneumonia when patients were nursed semirecumbent.
  - attempts must be made to keep not only the head of the bed elevated but also the patient's thorax.

FAST HUGS BID

- ## ULCER (STRESS) PROPHYLAXIS
  - **Notably for patients with**
    - Respiratory failure
    - Coagulation abnormalities
    - Steroid therapy
    - Hx. of gastroduodenal ulcer + risk of stress-related GI hemorrhage
- **No need for the routine use of antiulcer agents in all ICU** patients, including after trauma or major surgery.
- Antacids, Sucralfate, H2-antagonists, PPI (proton pump inhibitors); **the optimal medication is still NOT clear.**

FAST HUGS BID

GLYCEMIC CONTROL

- **Close glucose control**
  - the study of Van den Berghe et al.
  - the strict blood sugar levels of **80–110 mg/dL**
  - difficult to adhere to in routine patient care,
- **Now** aim to keep
  - blood sugar levels **below about 150 mg/dL**
  - recently published guidelines for the management of severe sepsis and septic shock

FAST HUGS BID

• SPONTANEOUS BREATHING TRIAL

- **Daily assessment of a SBT**; safe, effective, and highly predictive !!!
- **Prolonged mechanical ventilation**

  ➔ VAP ↑ , Mortality ↑ (in-hospital, total)
- A **SBT** should be …
  - at least daily
  - in a highly protocolized fashion
  - by a well trained team of nurses and respiratory therapists.

FAST HUGS BID

AJOU TRAUMA CENTER
경기남부 권역외상센터

B

- # BOWEL REGIMEN

  - Disorders of gastrointestinal motility, including **ileus, constipation, and diarrhea,** are common in critically ill patients and may contribute to additional disease burden.

- **Diarrhea** ➔ electrolyte imbalances, dehydration, hemorrhoidal irritation with resultant anemia, and delirium.
- **Constipation** ➔ patient discomfort, feeding intolerance, and delirium.
- **Institutional guidelines and use of standardized definitions**
- **Routine assessment and treatment to maintain normal bowel function** should be conducted in all critically ill patients.

FAST HUGS BID

INDWELLING CATHETER REMOVAL

- Arterial, CVC, Urinary, Dialysis catheters…
- Penetrate through the body's natural protective mechanisms ➔ local and systemic infections ↑
- Early discontinuation and removal
  ➔ catheter-associated infections ↓

- **Daily (or more frequent) assessment** should be performed of the ongoing need for these catheters, and their removal, when not medically necessary.

FAST HUGS BID

D

DE-ESCALATION OF ANTIBIOTICS

- Once a pathogen has been identified and antimicrobial susceptibilities have been reported, the regimen should be converted to **the most narrow-spectrum, cost-effective, and pathogen-specific antibiotic.**
- This practice, known as antibiotic de-escalation or streamlining, minimizes exposure to broad-spectrum antimicrobial therapy.

- The same principle of de-escalation can be applied to other pharmacologic treatments, which should be regularly re-evaluated for appropriate indications to minimize risk of adverse effects and medication errors.

SUMMARY & CONCLUSION

AJOU TRAUMA CENTER
경기남부 권역외상센터

- Optimization of feeding, analgesia, and sedation and reminders of certain prophylactic and therapeutic interventions are no longer the responsibility of the physician alone but are very much **a team effort**.
- Nurses, physiotherapists, and respiratory therapists **can all apply** a Fast Hug as they treat each patient and may question why a patient is not being fed, for instance, or is not receiving thromboembolic prophylaxis.
- Indeed, the essentials of patient care are less likely to be forgotten or overlooked when there are more people paying attention, and increasingly high quality patient care requires **good teamwork**.
